# Noninvasive prediction of insufficient biochemical response after ursodeoxycholic acid treatment in patients with primary biliary cholangitis based on pretreatment nonenhanced MRI

**DOI:** 10.1007/s00330-023-10080-w

**Published:** 2023-08-15

**Authors:** Yun Zhang, Xiaoli Fan, Bin Song, Yifeng Liu, Yidi Chen, Tianying Zheng, Yuxin Guo, Ting Duan, Zixing Huang, Li Yang

**Affiliations:** 1 Department of Radiology, West China Hospital, Sichuan University, No.37 Guoxue Alley, Wuhou District, Chengdu, 610041 Sichuan China; 2 Department of Gastroenterology and Hepatology, Sichuan University-University of Oxford Huaxi Joint Centre for Gastrointestinal Cancer, West China Hospital, Sichuan University, Chengdu, 610041 Sichuan China; 3 Department of Radiology, Sanya People’s Hospital, Sanya, Hainan China; 4 Department of Radiology, West China Tianfu Hospital of Sichuan University, Chengdu, China

**Keywords:** Biliary cirrhosis, Fibrosis, Ursodeoxycholic acid, Magnetic resonance, Cholangiopancreatography

## Abstract

**Objectives:**

To explore the feasibility of pretreatment nonenhanced magnetic resonance imaging (MRI) in predicting insufficient biochemical response to ursodeoxycholic acid (UDCA) in patients with primary biliary cholangitis (PBC).

**Methods:**

From January 2009 to April 2022, consecutive PBC patients who were treated with UDCA and underwent nonenhanced MRI within 30 days before treatment were retrospectively enrolled. All MR images were independently evaluated by two blinded radiologists. Uni- and multivariable logistic regression analyses were performed to develop a predictive model for 12-month insufficient biochemical response. Model performances were evaluated by computing the area under the receiver operating characteristic curve (AUC), sensitivity, and specificity.

**Results:**

A total of 74 patients (50.6 ± 11.9 years; 62 females) were included. Three pretreatment MRI features, including hepatomegaly (odds ratio [OR]: 4.580; *p* = 0.011), periportal hyperintensity on T2-weighted imaging (T2WI) (OR: 4.795, *p* = 0.008), and narrowing of the bile ducts (OR: 3.491; *p* = 0.027) were associated with 12-month insufficient biochemical response in the multivariable analysis. A predictive model based on the above indicators had an AUC of 0.781, sensitivity of 85.4%, and specificity of 61.5% for predicting insufficient biochemical response.

**Conclusions:**

A noninvasive model based on three pretreatment MRI features could accurately predict 12-month insufficient biochemical response to UDCA in patients with PBC. Early identification of PBC patients at increased risk for insufficient response can facilitate the timely initiation of additional treatment.

**Clinical relevance statement:**

A noninvasive predictive model constructed by incorporating three pretreatment MRI features may help identify patients with primary biliary cholangitis at high risk of insufficient biochemical response to ursodeoxycholic acid and facilitate the timely initiation of additional treatment.

**Key Points:**

*• Noninvasive imaging features based on nonenhanced pretreatment MRI may predict an insufficient biochemical response to UDCA in PBC patients.*

*• A combined model based on three MRI features (hepatomegaly, periportal hyperintensity on T2-weighted imaging, and narrowing of the bile ducts) further improved the predictive efficacy for an insufficient biochemical response to UDCA in PBC patients, with high sensitivity and specificity.*

*• The nomogram of the combined model showed good calibration and predictive efficacy for an insufficient biochemical response to UDCA in PBC patients. In particular, the calibration curve visualised the clinical applicability of the prediction model.*

**Supplementary Information:**

The online version contains supplementary material available at 10.1007/s00330-023-10080-w.

## Introduction

Primary biliary cholangitis (PBC) is an autoimmune-mediated intrahepatic cholestatic disease characterised by progressive, nonsuppurative, destructive intrahepatic cholangitis, leading to hepatic fibrosis and eventually cirrhosis and a range of complications [1]. According to the latest statistics from the 2022 Asia-Pacific Society for the Study of the Liver (APASL) guidelines [2], the global incidence and prevalence of PBC (17.6 per million people/year and 146 per million people/year, respectively) have exceeded previous estimates and are increasing at an incalculable rate, particularly notable in the Asia-Pacific region (8.4 and 98.2~118.8 per million, respectively) [3–6]. PBC has become a serious chronic liver disease threatening human health.

Ursodeoxycholic acid (UDCA) is the only first-line drug recommended by international guidelines and most countries for PBC treatment [7–9]. However, nearly 30~40% of PBC patients fail to achieve a sufficient therapeutic response and are at high risk of progressing to liver failure or liver transplantation [8, 10]. Previous studies have shown that PBC patients with an insufficient biochemical response have a significantly lower 10-year transplant-free survival rate than those with a sufficient biochemical response (51% vs. 90%) and that insufficient biochemical response is an independent risk factor for death or liver transplantation in PBC patients [11]. In addition, a strong correlation was also found between insufficient biochemical response and a range of complications of cirrhosis in PBC patients [12].

The guidelines recommend the use of biochemical response criteria to assess drug efficacy in PBC patients treated with UDCA in order to promptly identify insufficient responders and provide second-line therapy, such as obeticholic acid (OCA), fibrate, or budesonide to further improve the prognosis of such patients [8, 9]. Several criteria, including the Barcelona Definition, Paris I and Paris II criteria, and Toronto, Rotterdam, and POISE trial criteria, are currently available to evaluate the biochemical response to UDCA treatment in PBC patients [11, 13–15]. However, the above criteria all rely on the laboratory findings of patients 6 to 24 months after UDCA treatment. During this period, patients at high risk of an insufficient response to UDCA may experience rapid disease progression due to a lack of timely intervention. Thus, there is an urgent need to identify the characteristics or risk factors of PBC patients with insufficient response to UDCA, in order to achieve early screening of high-risk PBC patients prior to UDCA treatment and to assist in decision-making.

Magnetic resonance imaging (MRI), with its advantages of noninvasiveness, high soft tissue contrast, and multiparameter imaging, has long played an important role in the diagnosis, grading, and prognosis prediction of liver disease [16]. MR cholangiopancreatography (MRCP), a technique that uses heavy T2-weighted sequences to show tissue structures with very long T2 relaxation times, has significant advantages in showing bile duct lesions [17]. Previous studies [18–23] have explored the value of MRI in the diagnosis and staging of PBC and in the assessment of fibrosis. However, these studies have mainly focused on the diagnosis of PBC, and to our knowledge, no study has reported the potential of MRI in predicting the insufficient biochemical response to UDCA treatment in PBC patients.

Therefore, the purpose of this study was to explore the feasibility of applying pretreatment nonenhanced MRI to predict an insufficient biochemical response to UDCA treatment in patients with PBC.

## Materials and methods

### Ethics

This single-centre retrospective study was approved by the West China Hospital of Sichuan University Ethical Research Committee Centre, and the requirement for written informed consent was waived.

### Inclusion and exclusion criteria

From January 2009 to April 2022, consecutive patients who fulfilled the following inclusion criteria were included: (1) established diagnosis of PBC, (2) 13–15 mg/kg/d UDCA prescription, and (3) abdominal MRI examination (a 2D or 3D MRCP sequence was included in the scanning protocol) within 30 days before UDCA treatment.

The exclusion criteria were as follows: (1) comorbid with tumours or other causes of chronic liver disease such as viral hepatitis and alcoholic cirrhosis, or immune system diseases such as systemic lupus erythematosus; (2) combined with decompensated cirrhosis; (3) incomplete baseline clinical or laboratory indicators; (4) absence of follow-up information or follow-up duration of less than 12 months; (5) death during the follow-up period; and (6) poor image quality or incomplete abdominal MRI scans that could not be evaluated.

### Diagnostic criteria for PBC

The diagnosis of PBC was based on the criteria proposed in the 2018 Practice Guidance from the American Association for the Study of Liver Diseases (AASLD) [7]. A definitive diagnosis was established when two or more of the following three criteria were met: (1) cholestasis as indicated by elevated alkaline phosphatase (ALP); (2) the presence of anti-mitochondrial antibodies (AMAs) or PBC-specific autoantibodies such as sp100 or gp210, if the AMA test was negative; and (3) the presence of histological evidence supporting nonsuppurative destructive cholangitis and interlobular bile duct destruction.

### Clinical and laboratory data collection

All laboratory parameters were examined in the Department of Laboratory Medicine of West China Hospital, which was certified by the College of American Pathologists (CAP). The diagnosis of cirrhosis was made through imaging tests, including ultrasonography (US), computed tomography (CT), and/or MRI.

The clinical data included sex, age at diagnosis, presence of cirrhosis, dosage of UDCA, last date of follow-up, and UDCA response. The laboratory parameters included AMAs, anti-nuclear antibodies (ANAs), total bilirubin (TBIL), alanine aminotransferase (ALT), aspartate aminotransferase (AST), ALP, gamma-glutamyl transferase (GGT), albumin (ALB), and globulin (GLB). All patients underwent follow-up, including clinical and laboratory evaluations, every 1–3 months.

The clinical and laboratory data were retrieved from electronic medical records. L.Y.F. and G.Y.X. reviewed the data under the supervision of a specialist in gastroenterology/hepatology (F.X.L.) with > 8 years of experience in the field of autoimmune liver disease. The investigators who reviewed the clinical data were blinded to the imaging data. The normal values of biochemical results were based on the standards for common clinical biochemical tests proposed by the Health Care Commission of the People’s Republic of China (http://www.nhc.gov.cn/). Biochemical results were normalised by the upper limit of normal (ULN) of each laboratory to homogenise data interpretation.

### UDCA treatment and evaluation criteria for biochemical response

All patients received standard UDCA treatment for more than 12 months (a daily dose of 13–15 mg/kg of body weight). The response to UDCA treatment was evaluated according to the Paris I criteria (ALP level ≤ 3 × ULN, together with AST level ≤ 2 × ULN and a normal bilirubin level at 12 months) for cirrhotic PBC patients and the Paris II criteria (ALP and AST ≤ 1.5 × ULN with normal bilirubin at 12 months) for noncirrhotic patients [24].

### MRI examination

All MRI examinations were performed by using a 3.0-T (MAGNETOM Skyra, Siemens Healthineers; SIGNA™ Premier, GE) or 1.5-T (uMR588, Shanghai United Imaging Healthcare) MRI system equipped with a body coil. The MRI sequences and parameters are listed in Supplementary Table 1. Diffusion-weighted images were obtained by using a navigator-triggered technique at *b* values of 0, 50, and 800 s/mm^2^, and the corresponding apparent diffusion coefficient maps based on *b* values of 0 and 800 s/mm^2^ were reconstructed.

### Imaging evaluation

The MRI features evaluated in this study were based on previous reports. These features were as follows: (1) morphological features of the liver, including hepatomegaly [18], liver surface nodularity [18], smooth liver contour [20], and liver lobe redistribution [20]; (2) characteristics of the liver parenchyma, including liver parenchyma heterogeneity [18], parenchymal lace-like fibrosis [20], periportal halo sign [25], and periportal hyperintensity on T2WI [25]; (3) portal hypertension–related features, including splenomegaly [18], portosystemic collaterals [18], ascites [18], and minimal perihepatic effusion [20]; (4) characteristics of the bile ducts [20], including configuration of the bile ducts, narrowing or enlarging of the bile ducts, and oedema of the gallbladder wall [26]; and (5) lymphadenopathy[27]. Considering the risk of PBC progressing to cirrhosis or liver fibrosis, several imaging features associated with early cirrhosis and liver fibrosis were also included in this study for evaluation [28–31]. The definition and reference of each MRI feature are listed in Supplementary Table 2.

Two radiologists, one with 8 years of experience in abdominal imaging (Z.Y.) and one with 10 years of experience in abdominal imaging (C.Y.D.), blinded to all clinical and laboratory data independently analysed the nonenhanced MRI scans on a picture archiving and communication system (PACS). To resolve discrepancies between the two reviewers, all MR images were reassessed together with a third, more experienced radiologist (H.Z.X.) with more than 15 years of experience in abdominal MRI until a consensus was reached.

### Statistical analysis

Continuous variables, reported as the mean ± standard deviation or median (range), were compared by Student’s *t* test or the Mann–Whitney *U* test, where applicable. Categorical variables, expressed as counts and percentages, were compared by the chi-square test or Fisher’s exact test, where applicable.

The kappa statistic was computed to assess interobserver agreement. Univariable and multivariable logistic regression analyses were used to identify clinical and imaging predictors for 12-month insufficient biochemical response and to develop the predictive model. All variables with *p* < 0.1 at the univariable analyses were included in the multivariable analysis, which was performed using a forwards stepwise method. A nomogram was constructed to improve the model visualisation and utility based on the regression coefficients of the multivariable logistic regression analysis. The area under the receiver operating characteristic curve (AUC), sensitivities, and specificities were calculated to assess discriminative performances of each indicator and a combined model constructed by three indicators. Calibration curves were plotted to assess model calibration.

All statistical analyses were performed using SPSS Statistics (IBM, Version 25) and R software (R Foundation for Statistical Computing, version 3.2.5). A two-tailed *p* < 0.05 was considered statistically significant.

## Results

### Study population

**Fig. 1:**
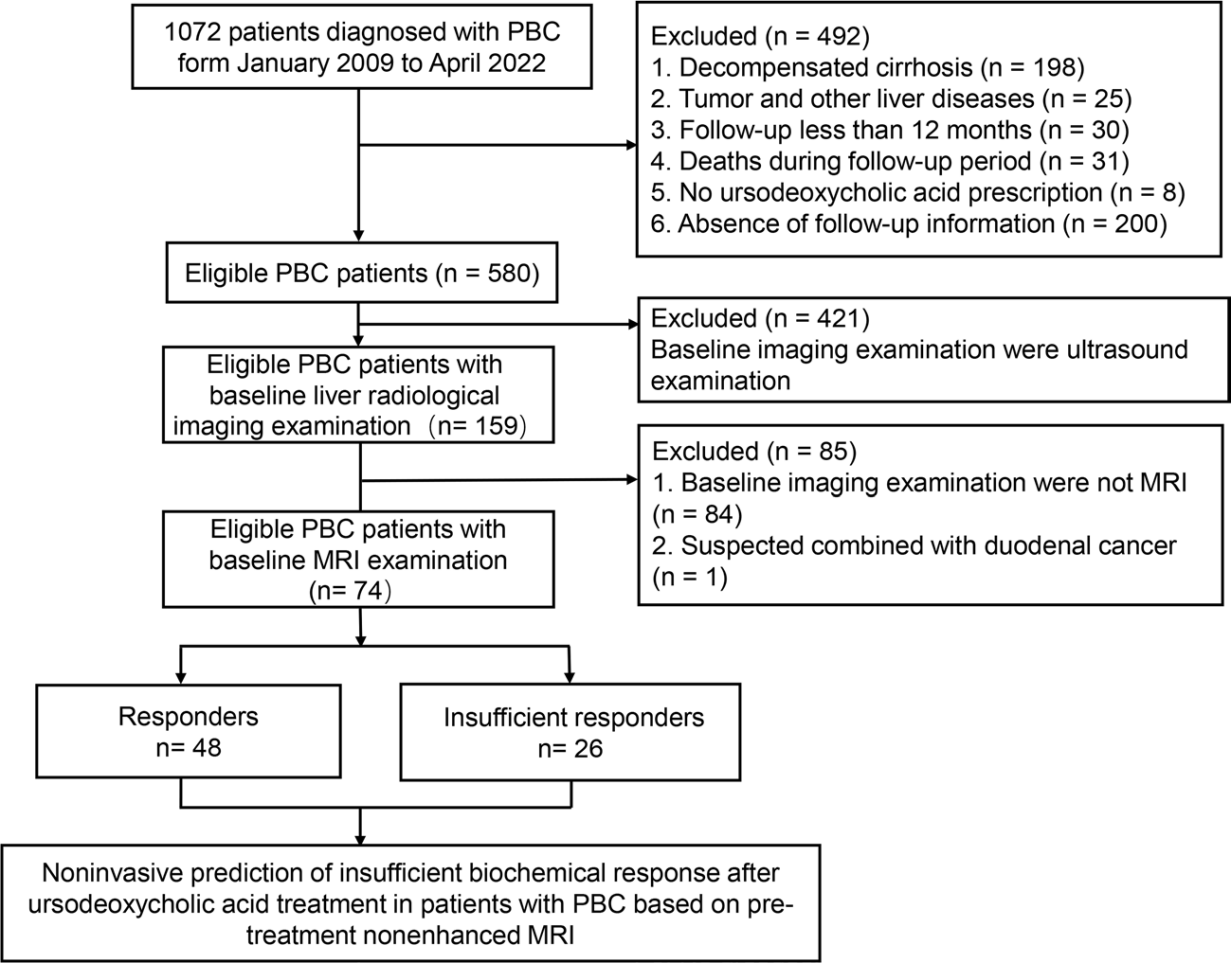
Flow chart of the study population. PBC, primary biliary cholangitis; MRI, magnetic resonance imaging

A total of 1072 consecutive patients who were diagnosed with PBC from January 2009 to April 2022 in our centre constituted the population database for this study. A total of 74 patients with PBC treated with UDCA who had a baseline abdominal MRI examination were finally included according to the inclusion and exclusion criteria (Fig. 1).

### Patient characteristics

Seventy-four patients (62 females; 83.8%) were included in this study, with an average age of 50.6 ± 11.6 years. Overall, 8 of the 74 (10.8%) patients had cirrhosis; regarding PBC-specific autoantibodies, 8 (10.8%) patients had AMA positivity, and 73 (98.6%) had ANA positivity. There were no significant differences between the sufficient responders and insufficient responders in terms of the presence of autoantibodies (*p* = 1.000) or cirrhosis (*p* = 1.000). However, male patients had a higher rate of sufficient biochemical response to UDCA than female patients (*p* = 0.047). Regarding laboratory parameters, insufficient responders showed higher levels of serological parameters at diagnosis, including TBIL (*p* = 0.012), ALT (*p* = 0.015), AST (*p* = 0.005), ALP (*p* < 0.001), and GGT (*p* = 0.011). The baseline clinical and laboratory characteristics of the patients with PBC are summarised in Table 1.

**Table 1:** Baseline clinical and laboratory characteristics of patients with PBC

Characteristics	Total* (*n* = 74)	Responder* (*n* = 48)	Insufficient responder* (*n* = 26)	*p* value
Patients’ characteristics				
Age^#^	50.6 ± 11.9	50.9 ± 14.0	50.0 ± 6.8	0.408
Sex				0.047
Male	12 (16.2)	11 (22.9)	1(3.8)	
Female	62 (83.8)	37 (77.1)	25 (96.2)	
Cirrhosis				1
Cirrhosis (−)	66 (89.2)	43 (89.6)	23 (88.5)	
Cirrhosis (+)	8 (10.8)	5 (10.4)	3 (11.5)	
AMA				1
AMA (−)	66 (89.2)	43 (89.6)	23 (88.5)	
AMA (+)	8 (10.8)	5 (10.4)	3 (11.5)	
ANA				1
ANA (−)	1 (1.4)	1 (2.1)	0 (0)	
ANA (+)	73 (98.6)	47 (97.9)	26 (100.0)	
Serum TBIL level (μmol/L)^#^	28.3 (4.9–324.5)	20.3 (6.0–130.0)	43.1 (4.9–324.5)	0.012
Serum ALT level (IU/L)^#^	80.7 (11.0–258.0)	74.3 (11.0–258.0)	92.6 (24.0–234.0)	0.015
Serum AST level (IU/L)^#^	77.4 (20.0–243.0)	69.4 (20–243)	92.3 (32.0–193.0)	0.005
Serum ALP level (IU/L)^#^	353.3 (69.0–1348.0)	281.9 (69–1348)	485.2 (210.0–1113.0)	< 0.001
Serum GGT level (IU/L)^#^	434.2 (20.0–2692.0)	362.7 (20–1525)	566.4 (148.0–2692.0)	0.011
Serum ALB level (IU/L)^#^	44.1 (31.5–53.7)	44.3 (32.3–53.7)	43.5 (31.5–50)	0.678
Serum GLB level (IU/L)^#^	33.5 (20.5–48.5)	33.7 (20.5–48.5)	33.1 (21.0–40.1)	0.968

*PBC*, primary biliary cholangitis; *AMA*, anti-mitochondrial antibody; *ANA*, anti-nuclear antibodies; *TBIL*, total bilirubin; *ALT*, alanine aminotransferase; *AST*, aspartate aminotransferase; *ALP*, alkaline phosphatase; *GGT*, gamma-glutamyl transferase; *ALB*, albumin; *GLB*, globulin

*Unless otherwise indicated, data are number of patients, with percentage in parentheses. Categorical variables were compared by using the chi-square test, Fisher exact test, or Kruskal–Wallis *H* test

^#^Data are continuous variables, reported as mean ± standard deviation or median (range), and were compared by using the two-sample *t* test or Mann–Whitney *U* test

### Baseline MRI features of patients

All patients underwent specialised imaging evaluations of five aspects: liver morphology, characteristics of the liver parenchyma, portal hypertension, characteristics of the bile ducts, and lymphadenopathy. The imaging characteristics of the bile ducts were evaluated on 2D or 3D MRCP images. The baseline MRI features of the patients are summarised in Table 2.

**Table 2:** Baseline MRI features of patients with PBC

Characteristics	Responder* (*n* = 48)	Insufficient responder* (*n* = 26)	*p* value
Liver morphology			
Hepatomegaly	11 (23.0)	13 (50)	0.022
Liver surface nodularity	43 (89.6)	23 (88.5)	1
Smooth liver contour	6 (12.5)	3 (11.5)	1
Right posterior hepatic notch	6 (12.5)	5 (19.2)	0.502
Widening of hepatic fissure	7 (14.6)	2 (7.6)	0.48
Periportal space widening	1 (2.1)	6 (23.0)	0.41
Expanded gall bladder fossa	0 (0)	0 (0)	...
Liver lobe redistribution	30 (62.5)	15 (57.7)	0.804
Liver parenchyma			
Liver parenchyma heterogeneous	45 (93.8)	24 (92.3)	1
Parenchymal lace-like fibrosis	19 (39.6)	7 (26.9)	0.317
Periportal halo sign	22 (45.8)	18 (69.2)	0.086
Periportal hyperintensity on T2WI	18 (37.5)	18 (69.2)	0.014
Portal hypertension			
Splenomegaly	7 (14.6)	11 (42.3)	0.011
Portosystemic collaterals	11 (22.9)	7 (26.9)	0.779
Portal vein dilatation	3 (6.25)	0 (0)	0.548
Ascites	32 (66.7)	16 (61.5)	0.799
Minimal perihepatic effusion	5 (10.4)	7 (26.9)	0.098
Characteristics of the biliary ducts			
Irregular of the biliary ducts	17 (35.4)	14 (53.8)	0.145
Narrowing of the biliary ducts	16 (33.3)	16 (61.5)	0.027
Enlarging of the biliary ducts	12 (25.0)	5 (19.2)	0.773
Oedema of the gallbladder wall	0 (0)	1 (3.8)	0.351
Lymphadenopathy	34 (43.6)	21 (80.8)	0.414

*MRI*, magnetic resonance imaging; *MRCP*, magnetic retrograde cholangiopancreatography; *PBC*, primary biliary cholangitis; *T2WI*, T2-weighted imaging

*Unless otherwise indicated, data are number of patients, with percentage in parentheses. Categorical variables were compared by using the chi-square test, Fisher exact test, or Kruskal–Wallis *H* test

**Fig. 2:**
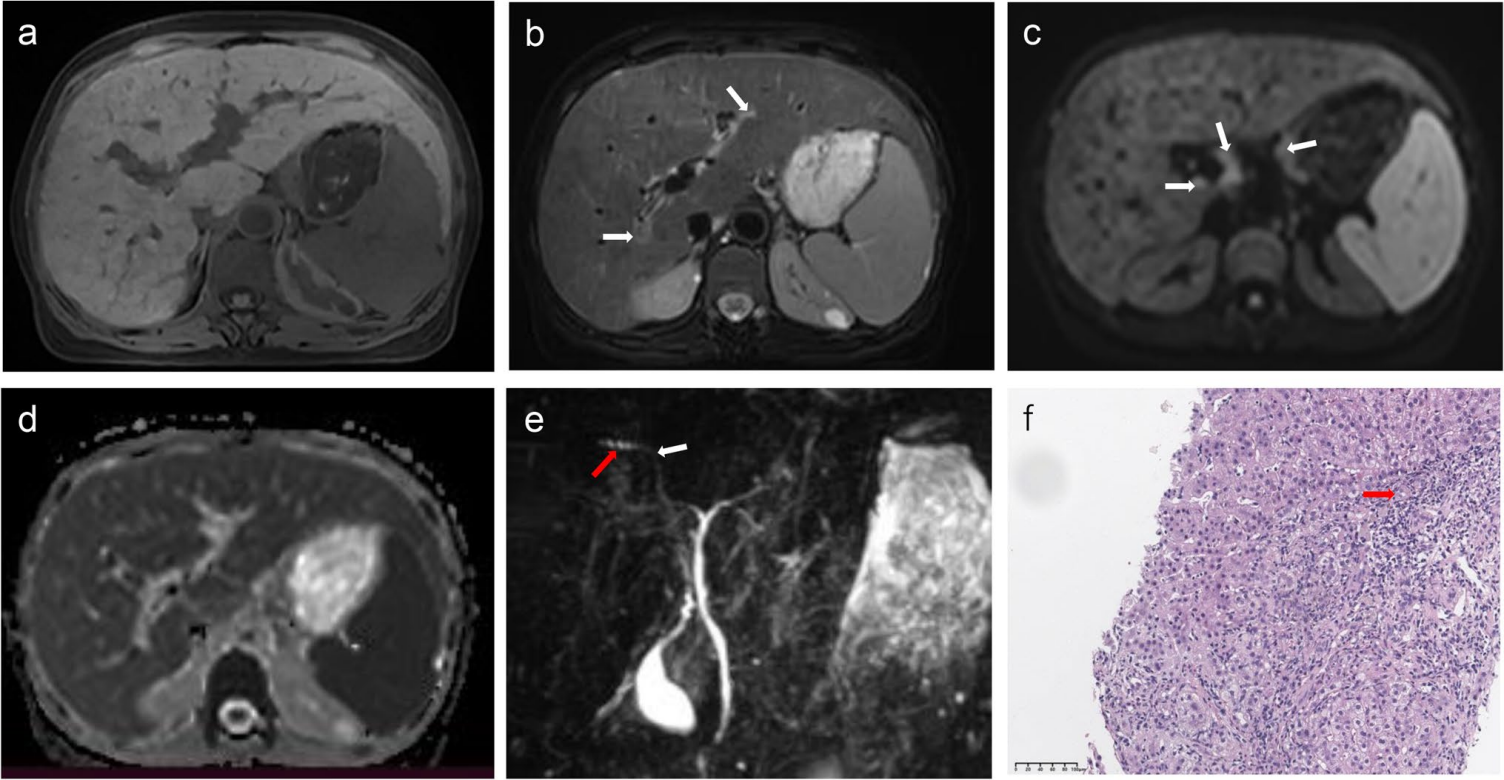
A 55-year-old female with AMA+ and ANA+ primary biliary cholangitis. T1-weighted imaging (**a**) shows hepatomegaly, splenomegaly, and liver surface nodularity; T2-weighted imaging (**b**) shows heterogeneity of the liver parenchyma and periportal hyperintensity (arrows); diffusion-weighted imaging (*b* value = 800 s/mm^2^) (**c**) shows lymphadenopathy in the hilar and hepatogastric ligament regions (arrows); the apparent diffusion coefficient map (**d**) shows an absence of diffusion restriction lesion; and magnetic resonance cholangiopancreatography (**e**) shows poor visualisation of small to medium intrahepatic bile ducts, and segmental irregularity and narrowing of the right intrahepatic bile duct (white arrow), with dilatation of the distal bile duct (red arrow). Haematoxylin-eosin (HE) staining at ×200 magnification (**f**) shows numerous infiltrating lymphocytes and plasma cells in the portal tract, a proliferation of poorly formed bile ductules (arrow) as well as ductopenia of interlobular bile duct. The patient was evaluated as having a 12-month insufficient biochemical response to UDCA by the Paris criteria I

**Fig. 3:**
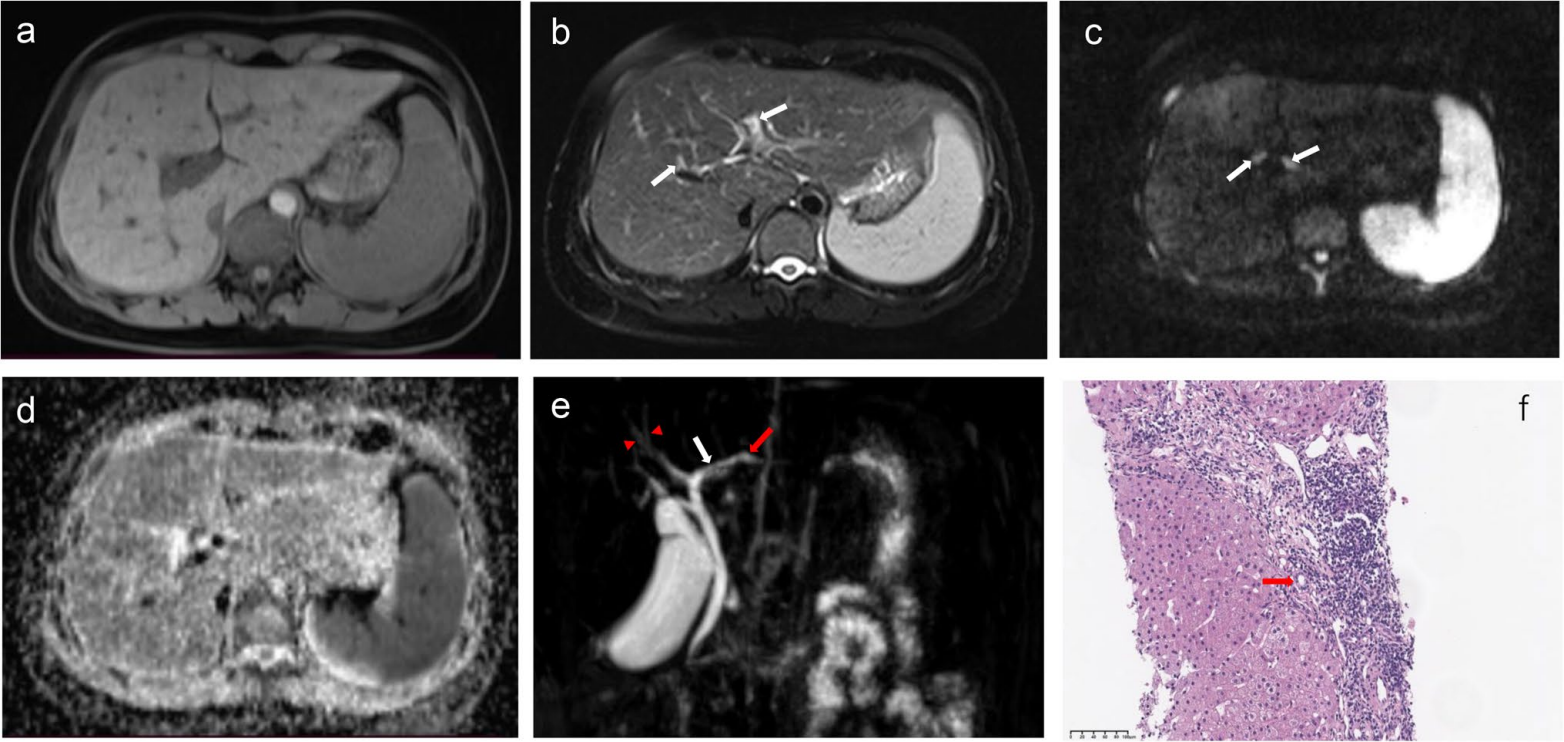
A 48-year-old female with AMA− and ANA+ primary biliary cholangitis. Axial T1-weighted imaging (**a**) shows mild liver surface nodularity and splenomegaly; T2-weighted imaging shows mild heterogeneity of the liver parenchyma and periportal hyperintensity (arrows); diffusion-weighted imaging obtained at *b* values of 800 s/mm^2^ (c) shows enlarged lymph nodes in the hepatic hilum; the apparent diffusion coefficient map shows no local diffusion restriction lesions (**d**); magnetic resonance cholangiopancreatography (**e**) shows irregularity and focal narrowing of the left intrahepatic bile duct (white arrow) and the enlargement of the distal bile duct (red arrow), and an irregular configuration of the right small to medium intrahepatic bile ducts (arrowheads). Haematoxylin-eosin (HE) staining at ×200 magnification (**f**) illustrates interface hepatitis and bile duct injury (arrow). The patient was evaluated having as 12-month insufficient biochemical response to UDCA by the Paris criteria II

**Fig. 4:**
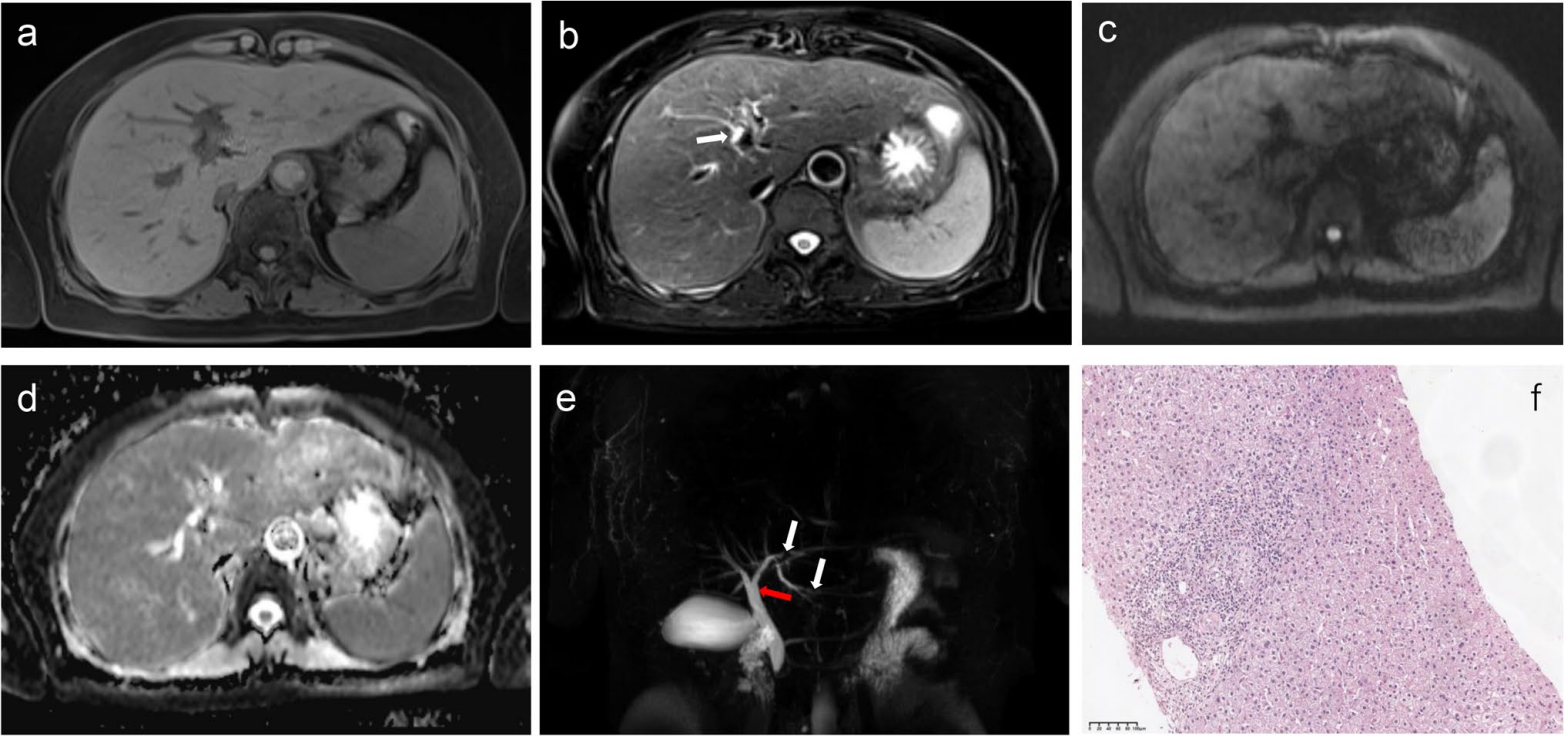
A 53-year-old female with AMA− and ANA+ primary biliary cholangitis. T1-weighted imaging (**a**) shows the liver with regular contour, T2-weighted imaging (**b**) shows heterogeneity of the liver parenchyma and periportal hyperintensity (arrow), diffusion-weighted imaging obtained at *b* values of 800 s/mm^2^ (**c**) and the corresponding apparent diffusion coefficient map (**d**) show no obvious abnormal diffusion restriction, and magnetic resonance cholangiopancreatography (**e**) shows irregularity and segmental narrowing of the medium to small left intrahepatic bile ducts (white arrows), and the dilatation of the common hepatic bile (red arrow). Haematoxylin-eosin (HE) staining at ×200 (**f**) magnification illustrates some infiltrating lymphocytes, monocytes, plasma cells, and neutrophils in the portal tract as well as bile duct loss. The patient was evaluated as 12-month insufficient biochemical response to UDCA by the Paris criteria I

Hepatomegaly (*p* = 0.022), periportal hyperintensity on T2WI (*p* = 0.014), and splenomegaly (*p* = 0.011) were significantly more frequent in the patients in the insufficient-response group. A higher proportion of patients in the insufficient-response group presented narrowing of the intrahepatic or extrahepatic bile duct (61.5% vs. 33.3%, *p* = 0.027) (Figs. 2, 3, and 4). The interobserver agreement between the two reviewers is summarised in Supplemental Table 3. Kappa coefficients were calculated for each MRI feature, ranging from 0.105 to 1.000. Of these, 14 of 22 (63.6%) MRI features had moderate to perfect interobserver agreement (0.4 ≤ *κ* ≤ 1.0).

### Prediction of biochemical response to UDCA

After 12 months of follow-up, 48 of 74 patients (64.9%) with PBC showed a sufficient biochemical response to UDCA treatment, and 26 (35.1%) patients with PBC showed an insufficient biochemical response. Sex, hepatomegaly, periportal halo sign, periportal hyperintensity on T2WI, splenomegaly, minimal perihepatic effusion, and narrowing of the bile ducts were identified as significant predictors of an insufficient biochemical response to UDCA in univariate analysis. However, only hepatomegaly (odds ratio [OR]: 4.580; *p* = 0.011), periportal hyperintensity on T2WI (OR: 4.795, *p* = 0.008), and narrowing of the bile ducts (OR: 3.491; *p* = 0.027) were significant predictors of insufficient biochemical response in multivariate analysis (Table 3).

**Table 3:** Univariate and multivariate analysis for predicting insufficient biochemical response after UDCA treatment in patients with PBC

Parameters	Univariate analysis			Multivariate analysis		
	OR	95% CI	*p* value	OR	95% CI	*p* value
Sex	7.432	0.902, 61.247	0.062	...	...	0.052
Serum TBIL level > 28 (μmol/L)	3.571	0.779, 16.365	0.101			
Serum ALT level > 40 (IU/L)	1.091	0.186, 6.397	0.923			
Serum AST level > 35 (IU/L)	2.907	0.321, 26.312	0.342			
Serum ALP level > 135 (IU/L)	...	...	0.999			
Serum GGT level > 45 (IU/L)	...	...	0.999			
Hepatomegaly	3.364	1.211, 9.345	0.02	4.58	1.408, 14.894	0.011
Liver surface nodularity	0.891	0.195, 4.063	0.882			
Smooth liver contour	0.913	0.209, 3.996	0.904			
Right posterior hepatic notch	1.667	0.455, 6.099	0.44			
Widening of hepatic fissure	0.488	0.094, 2.542	0.394			
Periportal space widening	0.28	0.032, 2.462	0.251			
Liver lobe redistribution	0.818	0.309, 2.165	0.686			
Liver parenchyma heterogeneous	0.8	0.125, 5.121	0.814			
Parenchymal lace-like fibrosis	0.562	0.198, 1.594	0.279			
Periportal halo sign	2.659	0.970, 7.286	0.057	...	...	0.096
Periportal hyperintensity on T2WI	3.75	1.356, 10.372	0.011	4.795	1.498, 15.157	0.008
Splenomegaly	4.295	1.406, 13.125	0.011	...	...	0.305
Portosystemic collaterals	1.239	0.414, 3.713	0.702			
Portal vein dilatation	...	...	0.999			
Ascites	0.8	0.297, 2.158	0.659			
Minimal perihepatic effusion	3.168	0.891, 11.263	0.075	...	...	0.133
Configuration of the biliary ducts	2.127	0.805, 5.622	0.128			
Narrowing of the biliary ducts	3.2	1.186, 8.631	0.022	3.491	1.153, 10.574	0.027
Enlarging of the biliary ducts	0.714	0.221, 2.310	0.574			
Oedema of the gallbladder wall	...	...	1			
Lymphadenopathy	1.729	0.544, 5.500	0.353			

Variables with *p* < 0.1 in univariate analysis were applied to multivariate analysis using a stepwise regression model (forward LR)

*UDCA*, ursodeoxycholic acid; *PBC*, primary biliary cholangitis; *TBIL*, total bilirubin; *ALT*, alanine aminotransferase; *AST*, aspartate aminotransferase; *ALP*, alkaline phosphatase; *GGT*, gamma-glutamyl transferase; *T2WI*, T2-weighted imaging; *OR*, odds ratio

**Fig. 5:**
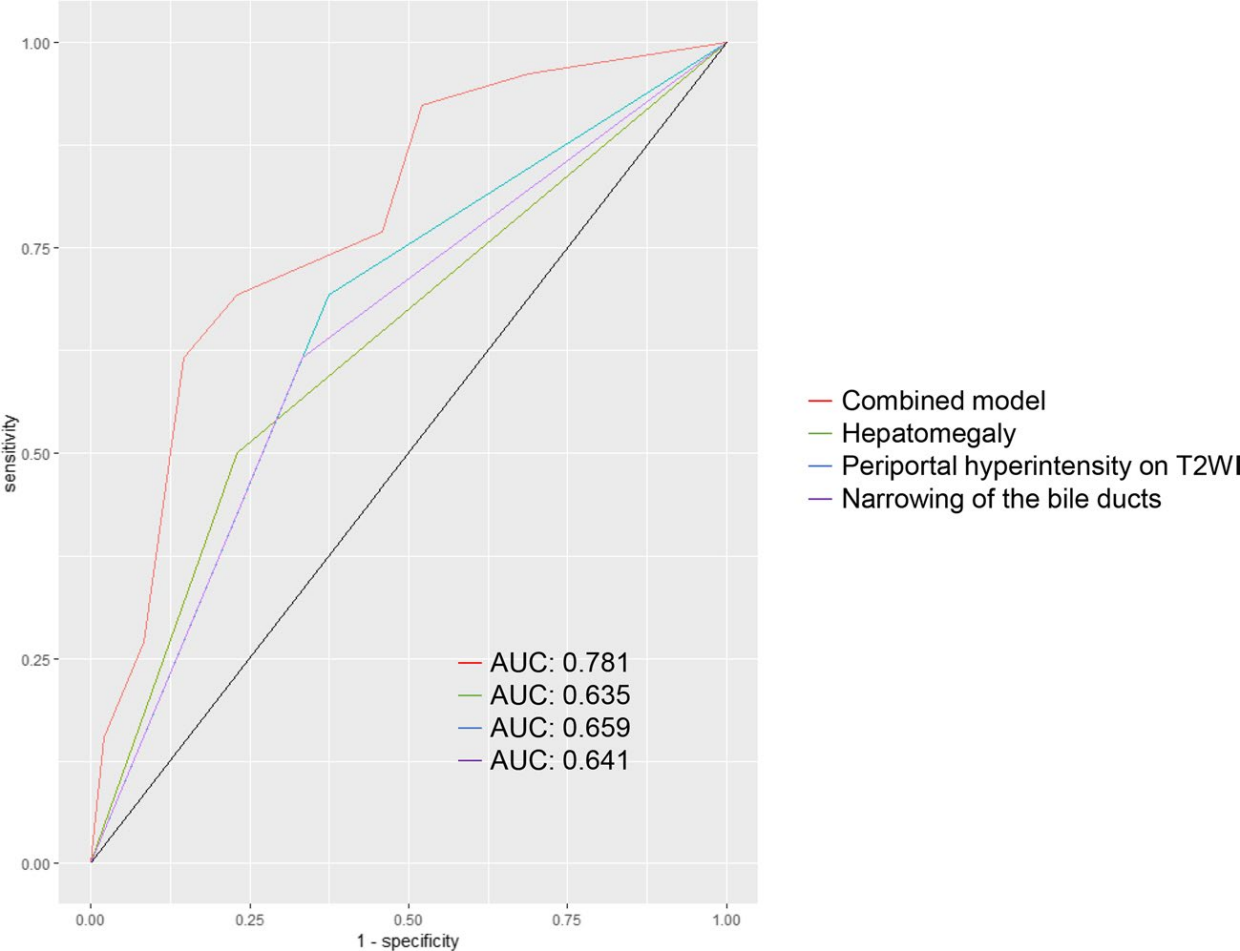
ROC curves showing the performance of individual indicators and the combined model in predicting an insufficient biochemical response to UDCA in PBC patients. The AUC, sensitivity, and specificity values of the individual indicators and combined model were as follows: hepatomegaly (0.635, 77.1%, 50%), periportal hyperintensity on T2WI (0.659; 62.5%, 30.8%), narrowing of the bile ducts (0.641, 66.7%, 38.5%), and the combined model (0.781, 85.4%, 61.5%)

ROC curves were utilised to compare the predictive efficacies of the individual predictors and the combined prediction model (the concomitant three MRI characteristics were present) (Fig. 5). The results showed that the combined prediction model had a good prediction efficiency. A predictive model based on the above indicators showed an AUC of 0.781, sensitivity of 85.4%, and specificity of 61.5% for predicting insufficient biochemical response (shown in Table 4).

**Table 4:** Diagnostic performance of significant indicators in prediction of insufficient biochemical response after UDCA treatment in patients with PBC

Characteristics	AUC	Sensitivity (%)	Specificity (%)
Hepatomegaly	0.635	77.1	50
Periportal hyperintensity on T2WI	0.659	62.5	30.8
Narrowing of the biliary ducts	0.641	66.7	38.5
Combined model	0.781	85.4	61.5

*UDCA*, ursodeoxycholic acid; *PBC*, primary biliary cholangitis; *T2WI*, T2-weighted imaging; *AUC*, area under the receiver operating characteristic curve

**Fig. 6:**
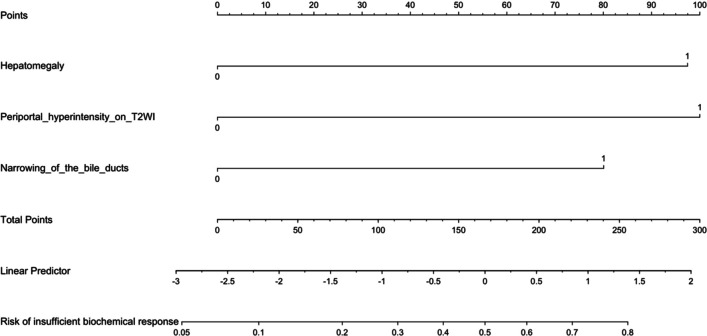
Nomogram based on the combined model to predict insufficient biochemical response to UDCA in PBC patients

A nomogram was constructed based on the combined model (Fig. 6). The calibration curve of the model, as a visual nomogram, for predicting the probability of an insufficient biochemical response demonstrated near-to-perfect alignment with the 45° line, indicating a good agreement between the prediction and observation. The H-L test showed a satisfactory goodness of fit (*p* = 0.6933) (Supplemental Figure 1).

## Discussion

Although UDCA has been recognised as the first-line treatment for PBC, up to 40% of patients show insufficient biochemical response to UDCA and are still at risk of progressing to advanced disease stages, such as liver fibrosis and cirrhosis. For this group of patients, the use of second-line therapies such as OCA, fibrate, or budesonide is expected to improve the prognosis and survival of patients [2]. However, assessing the biochemical response of PBC patients to UDCA using existing criteria requires a wait of 12 months or more after UDCA treatment. Thus, there is an urgent need for predictive indicators to screen those patients who are more likely to have an insufficient biochemical response to UDCA to facilitate early access to additional treatment. In this preliminary study, we found that the presence of three nonenhanced MRI features (hepatomegaly, periportal hyperintensity on T2WI, and narrowing of the bile ducts) was suggestive of an insufficient biochemical response to UDCA in PBC. The combined model based on three indicators showed a good predictive efficacy with high sensitivity and specificity. The nomogram based on this noninvasive model showed good calibration and predictive ability for predicting the risk of insufficient response to UDCA in PBC.

In our study, the feature of periportal hyperintensity on T2WI was significantly associated with an insufficient biochemical response after UDCA treatment in patients with PBC, and its OR value (OR: 4.795, *p* = 0.008) was the highest among the three risk factors. Periportal hyperintensity on T2WI is not unique to PBC and may be observed in all processes leading to periportal oedema, ductal hyperplasia, lymphatic vessel dilation, and inflammatory cell infiltration [20]. In PBC, this sign may be associated with active inflammation of the portal venous branches and interfacial hepatitis [32]. Previous studies have reported that patients with interfacial hepatitis are more likely to have an insufficient biochemical response [11, 15]. Our results may validate their findings. In addition, a controlled imaging-pathology study showed that periportal hyperintensity on T2WI was present in 85.7%, 66.7%, 66.7%, and 66.7% of PBC patients with stage 1, 2, 3, and 4 disease, respectively [19]. The decreased probability of this feature in advanced stages may be due to the replacement of the inflammatory process with the accumulation of fibrous tissue [33]. The presence of this feature may suggest that we need to closely monitor the follow-up images (e.g., disappearance of the feature) to determine whether the disease is progressing to an advanced stage.

Hepatomegaly is defined as an abnormal diffuse enlargement of the liver, but there is still a lack of consensus on the appropriate thresholds [34]. Imaging diagnosis of hepatomegaly is often based on the automatic acquisition on software of a workstation, and virtual value of liver volume can also be obtained based on calculation formulas [27, 35–37]. Nevertheless, to facilitate consideration of the volume of the liver in clinical practice, recent studies have commonly used unidimensional measurement as a diagnostic criterion for hepatomegaly in PBC [18, 19]. According to the results of previous studies, approximately 11 to 50% of patients with PBC present the feature of hepatomegaly, which varies depending on the stage of the disease [18, 27]. In the early stages of PBC, the liver tends to be normal in volume or mildly enlarged [27], whereas in patients with advanced stage or very severe portal hypertension, liver atrophy or segmental lobar hypertrophy or atrophy is more common [38, 39]. Our study found that hepatomegaly was a significant predictor of an insufficient response to UDCA in PBC. We speculate that this might be associated with damaged liver parenchymal cells, inflammatory cell infiltration, and liver stasis. The presence of hepatomegaly might suggest that the disease has started to progress from the earliest stages (normal liver volume) to the intermediate stages, and in patients treated with UDCA, an insufficient biochemical response may occur at this moment.

In addition, the presence of narrowing of the bile ducts was also associated with an insufficient biochemical response to UDCA in PBC. As reported in previous studies, in the early stages of PBC, when cirrhosis or liver fibrosis has not yet developed, the bile duct may appear normal on imaging in the vast majority of patients, while as the disease progresses towards cirrhosis, the intrahepatic bile ducts become destroyed and damaged, leading to fewer intrahepatic bile ducts and an irregular morphology [20]. Bergasa et al [40] reported that patients with fatigue or pruritus at diagnosis were at significantly higher risk of an insufficient UDCA response and were more likely to develop cirrhosis and its complications. Moreover, patients with advanced histology and bile duct deficiency were also reported to be more likely to show an insufficient biochemical response [11, 15]. Given these results, we hypothesise that patients with PBC who presented with narrowing of bile ducts are more likely to be in an advanced stage or have symptoms of liver function impairment. Therefore, treatment with UDCA at this stage may not be effective in improving patient prognosis.

We further developed a nomogram to visualise and validate the predictive power of the noninvasive model based on three MRI features. Compared with the findings of Tian et al [41] who developed a predictive model based on baseline clinical and laboratory indicators, the noninvasive imaging-based prediction model created in our work achieved a similar AUC (0.781 vs. 0.790) but with a higher sensitivity (85.4% vs. 80.4%). This may indicate that imaging can reflect pathological changes associated with the disease process earlier than laboratory markers.

Despite the relatively small sample size of this study, the results are exciting. Previous studies have mainly focused on the biochemical response in PBC patients by clinical symptoms, pathological staging, and biochemical indicators [11, 15, 40, 42]. To our knowledge, this study was the first to use pretreatment MRI features to predict the insufficient biochemical response to UDCA in PBC patients. Our study has good generalizability and application prospects. On the one hand, the selection of pretreatment indicators can help in the screening of high-risk patients with a poor response so that early interventions can be implemented. On the other hand, the predictive indicators proposed in our study are based on nonenhanced MRI examinations, which are simple, easy to master, economical to use in practice, and free from risks such as contrast allergy.

This study had several limitations. First, substantial selection bias could have been introduced due to the retrospective nature of the study. Second, due to the limited sample size (most patients underwent only ultrasound without MRI examination before UDCA treatment), in future studies, we would like to prospectively include a large sample size to find more stable noninvasive indicators that can effectively predict the biochemical response to UDCA therapy in PBC patients before changes in serological indicators occur, such as 6 months or earlier. Finally, this study was a retrospective case-control study, which might not accurately reflect real clinical conditions. Further large-scale multicentre studies are warranted to validate our model.

In conclusion, a noninvasive model based on pretreatment nonenhanced MRI features could predict an insufficient biochemical response in PBC patients after UDCA treatment. A nomogram based on potential risk factors may be used as a tool for clinicians to screen out patients who are not responding well to UDCA in advance so that early interventions can be implemented.

### Supplementary Information

ESM 1 (DOCX 460 kb)

## Abbreviations

2D: Two-dimensional
3D: Three-dimensional
AASLD: American Association for the Study of Liver Diseases
ALB: Albumin
ALP: Alkaline phosphatase
ALT: Alanine aminotransferase
AMA: Anti-mitochondrial antibody
ANA: Anti-nuclear antibody
APASL: Asia-Pacific Society for the Study of the Liver
AST: Aspartate aminotransferase
CAP: College of American Pathologists
GGT: Gamma-glutamyl transferase
GLB: Globulin
OCA: Obeticholic acid
OR: Odds ratio
PACS: Picture archiving and communication system
PBC: Primary biliary cholangitis
SSFSE: Single-shot fast spin–echo
T2WI: T2-weighted imaging
TBIL: Total bilirubin
UDCA: Ursodeoxycholic acid
ULN: Upper limit of normal

## Declarations

### Guarantor

The scientific guarantor of this publication is Dr. Li Yang.

### Statistics and biometry

No complex statistical methods were necessary for this paper.

### Methodology

- retrospective
- diagnostic or prognostic study
- performed at one institution
