# Supplementary material for: Noninvasive prediction of insufficient biochemical response after ursodeoxycholic acid treatment in patients with primary biliary cholangitis based on pretreatment nonenhanced MRI
Source: Eur Radiol. 2023 Aug 15;34(2):1268–79. doi: 10.1007/s00330-023-10080-w (PMC10853298; doi:10.1007/s00330-023-10080-w)

**Noninvasive prediction of insufficient biochemical response after ursodeoxycholic acid treatment in patients with primary biliary cholangitis based on pretreatment nonenhanced MRI**

**ELECTRONIC SUPPLEMENTARY MATERIAL**

**Contents:**

Supplementary-Table 1………………………………………………………………………………………………………….......2

Supplementary-Table 2………………………………………………………………………………………………………………3

Supplementary-Table 3………………………………………………………………………………………………………………6

Supplementary-Figure 1……………………………………………………………………………………………………………...7

| **Supplementary Table 1: MRI scan models, sequences, and parameters** | | | | | | | | | |
| --- | --- | --- | --- | --- | --- | --- | --- | --- | --- |
| **Sequence** | **Fat suppression** | **TR** | **TE** | **Flip angle (°)** | **ST** | **Spacing** | **Matrix**  **size** | **FOV** | **Acquisition Time**  **(s)** |
|  |  | **(ms)** | **(ms)** |  | **(mm)** | **(mm)** |  | **(mm2）** |  |
| **Siemens MAGNETOM Skyra 3.0 Tesla (18-channel body array coil)** | | | | | | | | | |
| T2-weighted 2D FSE | Yes | 2160 | 100 | 160 | 6 | 1.8 | 320×288 | 433×433 | 36 |
| Diffusion-weighted imaging* | Yes | 5600 | 68 | 90 | 6 | 1.8 | 100×76 | 380×289 | 233 |
| MRCP-2D-SSFSE | Yes | 4500 | 709 | 180 | 40 | - | 384×269 | 300×300 | 14 |
| MRCP-3D-breath hold | Yes | 2000 | 495 | 105 | 1.2 | 0 | 320×224 | 320×320 | 18 |
| In- and opposed-phase T1-weighted imaging | No | 81 | 2.72/1.4 | 70 | 6 | 1.8 | 352×286 | 400×325 | 24 |
| **GE SIGNA™ Premier 3.0 Tesla (30-channel body anterior coil)** | | | | | | | | | |
| T2-weighted 2D FSE | Yes | 2200 | 85 | 111 | 7 | 2 | 320×224 | 304×380 | 47 |
| Diffusion-weighted imaging* | Yes | 5000 | 62 | 90 | 7 | 2 | 120×240 | 380×380 | Respiratory gating |
| MRCP-2D-SSFSE | Yes | 5100 | 681.6 | 90 | 50 | - | 320×256 | 350×350 | 25 |
| MRCP-3D-breath hold | Yes | 2175 | 851 | 90 | 8 | 0 | 352×224 | 350×350 | 21 |
| In- and opposed-phase T1-weighted imaging | No | 146.8 | 2.3/1.1 | 55 | 7 | 2 | 320×192 | 342×380 | 16 |
| **uMR588 1.5 Tesla (6-channel body anterior coil)** | | | | | | | | | |
| T2-weighted 2D FSE | Yes | 2600 | 99.2 | 90 | 6.5 | 1.5 | 256×168 | 427×320 | 39 |
| Diffusion-weighted imaging* | Yes | 3350 | 77 | 90 | 6.5 | 10 | 128×92 | 320×400 | Respiratory gating |
| MRCP-2D-SSFSE | Yes | 4500 | 754.5 | 150 | 40 | - | 320×288 | 350×350 | 23 |
| MRCP-3D-breath hold | Yes | 1300 | 283.22 | 15 | 6 | 0 | 256×192 | 350×350 | 19 |
| In- and opposed-phase T1-weighted imaging | No | 117.6 | 2.27/1 | 60 | 6.5 | 1.3 | 256×174 | 320×400 | 29 |
| MRI: magnetic resonance imaging; TR: repetition time; TE: echo time; ST: section thickness; FOV: field of view; 2D: two-dimensional; FSE: fast spin-echo; SSFSE: single shot fast spin-echo; 3D: three-dimensional. * Images were acquired under free breath. | | | | | | | | | |

| **Supplementary Table 2. Qualitative and quantitative MR imaging characteristics of patients with PBC** | | | |
| --- | --- | --- | --- |
| **Imaging characteristics** | **Definition** | **References** | **Characteristic properties** |
| Hepatomegaly | Defined as craniocaudal diameter in medioclavicular line ≥ 15.5 cm | Idilman | Reported in previous studies as a characteristic imaging presentation of PBC |
| Liver surface nodularity | Irregular liver contour with blunted edge | Idilman |
| Smooth liver contour | Regular liver contour with smooth edge | Haliloglu |
| Liver lobe redistribution | Disproportion of liver lobes due to segmental hepatic atrophy/hypertrophy | Haliloglu |
| Liver parenchyma heterogeneous | Heterogeneity of the parenchyma was present if there was a  patchy or focal parenchymal signal abnormality | Idilman |
| Parenchymal lace-like fibrosis | Defined as ill-defined hyperintense areas on T2WI | Haliloglu |
| Periportal halo sign | Low-signal intensity centered around portal venous branches, 5 mm–1 cm in size with no mass effect on T2WI and/or T1WI | Wenzel |
| Periportal hyperintensity on T2WI | Hyperintensity around portal venous branches on T2WI | Wenzel |
| Splenomegaly | Defined as craniocaudal diameter ≥ 13 cm | Idilman |
| Portosystemic collaterals | Increased number and size of vessels around splenic hilum, paraesophageal region, and gastrohepatic ligament | Idilman |
| Minimal perihepatic effusion | Only a small amount of free fluid confined to the perihepatic area | Haliloglu |
| Configuration of the biliary ducts | Regular or irregular morphology of the biliary ducts | Haliloglu |
| Narrowing of the bile ducts | Focal narrowing of the intrahepatic bile ducts or common bile ducts | Haliloglu |
| Enlarging of the bile ducts | Focal or total dilated of the intrahepatic bile ducts or common bile ducts | Kovač |
| Ascites | Free fluid in abdomen or pelvis | Idilman |
| Lymphadenopathy | Defined as ≥ 2 nodes of ≥ 1 cm in the short axis | Blachar |
| Widening of hepatic fissure | Subjective widening of fissures for ligamentum teres and ligamentum venosum | Cannell | Reported in previous studies as a characteristic imaging presentation of cirrhosis or liver fibrosis |
| Periportal space widening | Enlarged (> 10 mm) perihilar periportal space measuring from the anterior margin of the right portal vein to the posterior margin of the left medial segment | Ludwig |
| Right posterior hepatic notch | Sharp notch in the posterior-inferior liver surface | Venkatesh |
| Expanded gall bladder fossa | Enlargement of the peri-cholecystic space, bounded laterally by the right hepatic lobe and medially by the left lateral segment | Venkatesh |
| Portal vein dilatation | defined as portal vein diameter > 13 mm | Zhang |
| Edema of the gallbladder wall | Edematous thickening of the gallbladder wall with high signal on T2WI | Khoshpour | Reported in previous studies as a characteristic imaging presentation of primary sclerosing cholangitis |
| MR: magnetic resonance; PBC: primary biliary cholangitis; T2WI: T2-weighted imaging; T1WI: T1-weighted imaging | | | |

**References:**

1. Idilman IS, Venkatesh SH, Eaton JE, Bolan CW, Osman KT, Maselli DB et al. Magnetic resonance imaging features in 283 patients with primary biliary cholangitis. European radiology 2020;30:5139-5148

2. Haliloglu N, Erden A, Erden I. Primary biliary cirrhosis: Evaluation with t2-weighted mr imaging and mr cholangiopancreatography. European journal of radiology 2009;69:523-527

3. Wenzel JS, Donohoe A, Ford KL, 3rd, Glastad K, Watkins D, Molmenti E. Primary biliary cirrhosis: Mr imaging findings and description of mr imaging periportal halo sign. AJR. American journal of roentgenology 2001;176:885-889

4. Kovač JD, Ješić R, Stanisavljević D, Kovač B, Banko B, Seferović P et al. Integrative role of mri in the evaluation of primary biliary cirrhosis. European radiology 2012;22:688-694

5. Blachar A, Federle MP, Brancatelli G. Primary biliary cirrhosis: Clinical, pathologic, and helical ct findings in 53 patients. Radiology 2001;220:329-336

6. Cannella R, Dasyam N, Seo SH, Furlan A, Borhani AA. Performance of morphologic criteria for the diagnosis of cirrhosis in patients with non-alcoholic steatohepatitis compared to other etiologies of chronic liver disease: Effect of level of training and experience. Abdominal radiology (New York) 2021;46:960-968

7. Ludwig DR, Fraum TJ, Ballard DH, Narra VR, Shetty AS. Imaging biomarkers of hepatic fibrosis: Reliability and accuracy of hepatic periportal space widening and other morphologic features on mri. AJR. American journal of roentgenology 2021;216:1229-1239

8. Venkatesh SK, Yin M, Takahashi N, Glockner JF, Talwalkar JA, Ehman RL. Non-invasive detection of liver fibrosis: Mr imaging features vs. Mr elastography. Abdominal imaging 2015;40:766-775

9. Zhang Y, Zhang XM, Prowda JC, Zhang HL, Sant'anna Henry C, Shih G et al. Changes in hepatic venous morphology with cirrhosis on mri. Journal of magnetic resonance imaging : JMRI 2009;29:1085-1092

10. Khoshpouri P, Habibabadi RR, Hazhirkarzar B, Ameli S, Ghadimi M, Ghasabeh MA. Imaging features of primary sclerosing cholangitis: From diagnosis to liver transplant follow-up. 2019;39:1938-1964

| **Supplementary Table 3.**  **The kappa analysis of MR features between two reviewers.** | | | |
| --- | --- | --- | --- |
| Characteristics | Reviewer 1 | Reviewer 2 | kappa value |
| **Liver morphology** |  |  |  |
| Hepatomegaly | 24 (32.4) | 28 (37.8) | 0.704 |
| Liver surface nodularity | 66 (89.2) | 49 (66.2) | 0.329 |
| Smooth liver contour | 9 (12.2) | 19 (25.7) | 0.487 |
| Right posterior hepatic notch | 11 (14.9) | 9 (12.2) | 0.538 |
| Widening of hepatic fissure | 9 (12.2) | 13 (17.6) | 0.575 |
| Periportal space widening | 9 (12.2) | 9 (12.2) | 1.000 |
| Expanded gall bladder fossa | 0 (0.0) | 0 (0.0) | 1.000 |
| Liver lobes redistribution | 43 (58.1) | 34 (45.9) | 0.430 |
| **liver parenchyma** |  |  |  |
| Liver parenchyma heterogeneous | 69 (93.2) | 68 (91.9) | 0.313 |
| Parenchymal lace-like fibrosis | 26 (35.1) | 23 (31.1) | 0.117 |
| Periportal halo sign | 40 (54.1) | 40 (54.1) | 0.456 |
| Periportal hyperintensity on T2WI | 36 (48.6) | 46 (62.2) | 0.517 |
| **Portal hypertension** |  |  |  |
| Splenomegaly | 18 (24.3) | 21 (28.4) | 0.687 |
| Portosystemic collaterals | 18 (24.3) | 13 (17.6) | 0.311 |
| Portal vein dilatation | 3 (4.1) | 8 (10.8) | 0.517 |
| Ascites | 48 (64.9) | 35 (47.3) | 0.105 |
| Minimal perihepatic effusion | 12 (16.2) | 8 (10.8) | 0.196 |
| **Characteristics of biliary ducts** |  |  |  |
| Irregular of the biliary ducts | 31 (42.9) | 32 (43.2) | 0.364 |
| Narrowing of the biliary ducts | 32 (43.2) | 36 (48.6) | 0.566 |
| Enlarging of the biliary ducts | 17 (23.0) | 29 (39.2) | 0.510 |
| Edema of the gallbladder wall | 1 (1.4) | 2 (2.7) | 0.661 |
| **Lymphadenopathy** | 55 (74.3) | 43 (58.1) | 0.354 |
| MRI: magnetic resonance imaging; T2WI: T2-weighted imaging  * Inter-rater agreement was considered poor (κ < 0.2), fair (κ: 0.2-0.4), moderate (κ: 0.4-0.6), substantial (κ: 0.6-0.8) or almost perfect (κ: 0.8-1.0), respectively. | | | |

**Supplementary Figure 1:** The calibration curve of the nomogram based on the model for predicting the probability of an insufficient biochemical response demonstrated nearly perfect alignment with the 45° line, indicating good agreement between the prediction and observation. The H-L test showed a satisfactory goodness of fit (p = 0.6933). The C-index for the prediction nomogram was 0.7808 (95% CI: 0.672, 0.8897).


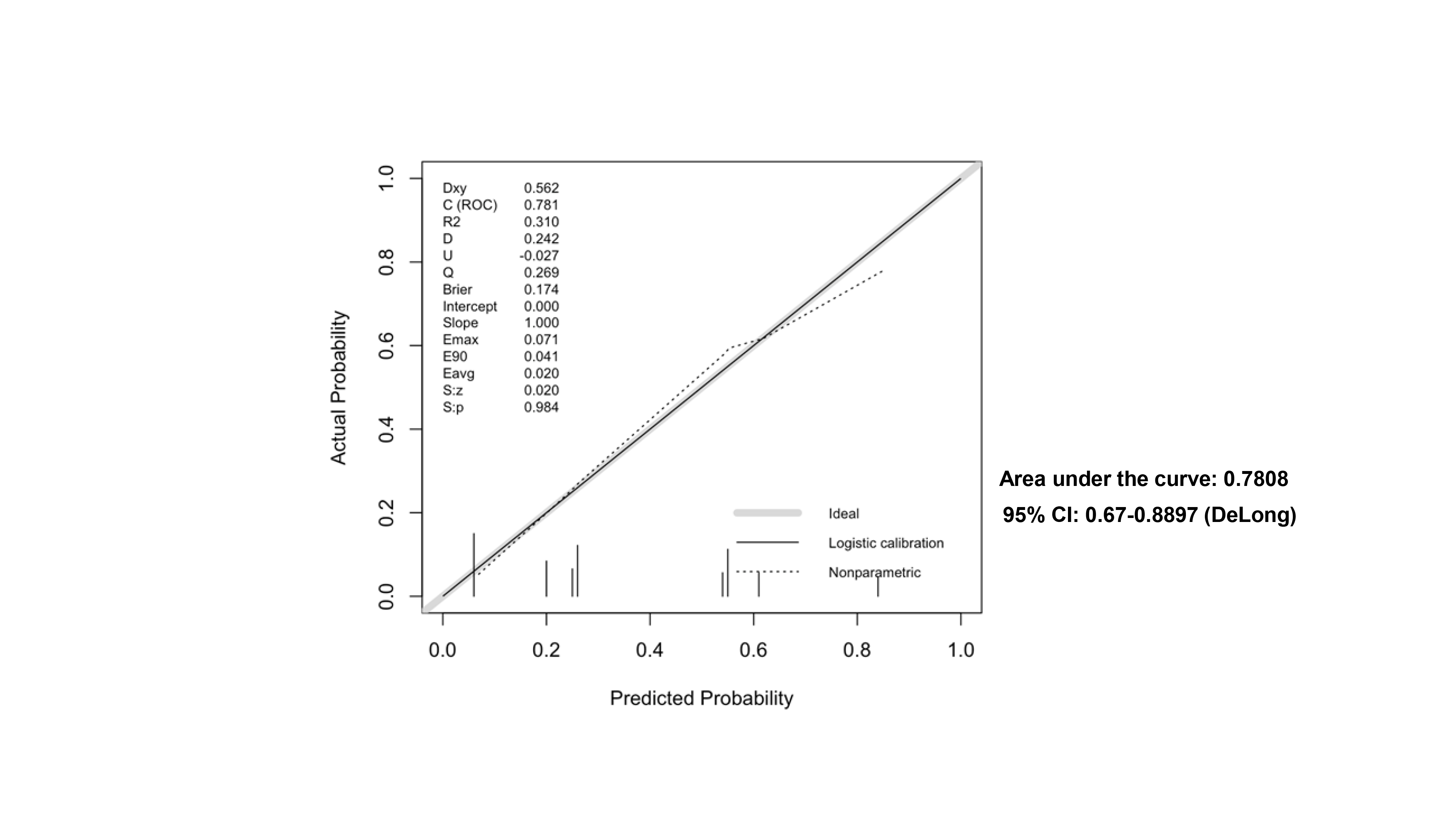

Supplement: Supplementary file 1 — (DOCX 460 kb) [file 330_2023_10080_MOESM1_ESM.docx]
